# Promotion of growth and metal accumulation of alfalfa by coinoculation with *Sinorhizobium* and *Agrobacterium* under copper and zinc stress

**DOI:** 10.7717/peerj.6875

**Published:** 2019-05-07

**Authors:** Liru Jian, Xiaoli Bai, Hui Zhang, Xiuyong Song, Zhefei Li

**Affiliations:** 1 State Key Laboratory of Crop Stress Biology in Arid Areas, Northwest A&F University, Yangling, Shaanxi, China; 2 Shaanxi Key Laboratory of Agricultural and Environmental Microbiology, College of Life Science, Northwest A&F University, Yangling, Shaanxi, China

**Keywords:** Copper and zinc stress, Co-inoculation, Enhance, Plant growth

## Abstract

The Legume-Rhizobium symbiosis has been proposed as a promising technique for the phytoremediation of contaminated soils due to its beneficial activity in symbiotic nitrogen fixation. However, numerous studies have shown that excessive heavy metals reduce the efficiency of symbiotic nodulation with Rhizobium and inhibit plant growth. In this study, we aimed to evaluate the synergistic effects of IAA-producing bacteria and Rhizobium on* Medicago lupulina* growth under Cu and Zn stress. Pot experiments showed that 400 mg kg^−1^ Cu^2 +^ and Zn^2 +^ greatly inhibited plant growth, but dual inoculation of *Medicago lupulina* with *Sinorhizobium meliloti* CCNWSX0020 and* Agrobacterium tumefaciens* CCNWGS0286 significantly increased the number of nodules and plant biomass by enhancing antioxidant activities. Under double stress of 400 mg kg^−1^ Cu^2 +^ and Zn^2 +^, the nodule number and nitrogenase activities of dual-inoculated plants were 48.5% and 154.4% higher, respectively, than those of plants inoculated with *Sinorhizobium meliloti*. The root and above-ground portion lengths of the dual-inoculated plants were 32.6% and 14.1% greater, respectively, than those of the control, while the root and above-ground portion dry weights were 34.3% and 32.2% greater, respectively, than those of the control. Compared with *S. meliloti* and *A. tumefaciens* single inoculation, coinoculation increased total Cu uptake by 39.1% and 47.5% and increased total Zn uptake by 35.4% and 44.2%, respectively, under double metal stress conditions. Therefore, coinoculation with *Sinorhizobium meliloti* and *Agrobacterium tumefaciens* enhances metal phytoextraction by increasing plant growth and antioxidant activities under Cu/Zn stress, which provides a new approach for bioremediation in heavy metal-contaminated soil.

## Introduction

Pollution of the biosphere by heavy metals, such as copper and zinc, has increased dramatically since industrial production and extensive use of chemical fertilizers and pesticides, as well as the use of industrial waste waters for irrigation (Ampofo & Awortwe, 2017; Malinowska & Jankowski, 2017). Although copper and zinc are essential trace elements for most living organisms, as they participate in electron transport, redox, and other metabolic reactions, excess copper or zinc could induce various morphological, physiological, and biochemical dysfunctions directly or indirectly in organisms. The most frequent and common consequence of copper or zinc toxicity to cells is to produce excessive reactive oxygen species (ROS). ROS can disrupt the redox status of cells and cause oxidative stress, leading to lipid peroxidation, membrane dismantling and damage to DNA, proteins and carbohydrates (Ajina et al., 2017; Zhang et al., 2016). It is necessary to remove excessive copper or zinc from contaminated soil. Many methods such as immobilization through pH alterations, removal, sequestration, and phytoextraction have been tested to extract heavy metal pollutants from contaminated soil (Saavedra et al., 2018; Bano et al., 2018; Huang et al., 2016).

In these methods, the unique ability of plants to remediate heavy metals has been widely investigated. Phytoremediation is using plants to take up heavy metals from the soil and accumulate metals in their tissue (Mleczek et al., 2017; Rezania et al., 2015). However, plants are not necessarily tolerant and may be subject to metal toxicity at high metal concentrations, leading to low biomass and poor remediation efficiency in high-concentration heavy metal-contaminated soil (Ebbs & Kochian, 1997). Therefore, it is a great challenge to search for a way to promote plant growth in heavy metal-contaminated soil. Rhizospheric microorganisms play an important role in plant nutrition, mineral dissolution and the production of plant growth-promoting substances (Paungfoolonhienne et al., 2014; Tashi-Oshnoei, Harighi & Abdollahzadeh, 2017). If plant biomass is increased, their net capacity to extract metals from soil is also improved; hence, better growth of plants greatly improves phytoremediation efficiency. Fatima and Ahmed found that *Bacillus cereus* significantly reduced the deleterious effects of Cr and promoted the growth of *Lens culinaris* growing in a chromium-contaminated environment (Fatima & Ahmed, 2018). In addition, several attempts have been made to illustrate the importance of endophytic bacteria on plant growth promotion and phytoremediation (Kong et al., 2017; Kuramshina, Smirnova & Khairullin, 2018; Ali et al., 2017). Among these plant growth-promoting rhizobacteria (PGPR), N_2_-fixing soil bacteria, namely, Rhizobia, are well known for their ability to establish symbiotic associations with legumes and develop into the structures called root nodules (Barauna et al., 2016). Thus, the nitrogenase complex catalyzes the ATP-dependent reduction of N_2_ to ammonium in root nodules (Khadka et al., 2017). Legume plants-Rhizobium symbiotic systems play a key role in enhancing the nitrogen pool of soil, leading to an increase in biomass and accumulation of heavy metals in contaminated soil (Hao et al., 2014; Pajuelo et al., 2011). The dry weight and nitrogen content of peas inoculated with *Rhizobium sp*. Rp15, isolated from heavy metal-polluted soil, were more than those of the control group when they grew in the Ni^2+^- and Zn^2+^-contaminated soil (Wani, Khan & Zaidi, 2008). Many environmental conditions, such as drought stress (Staudingera et al., 2016), extreme temperature (Ryalls et al., 2013; Peltzer, Abbott & Atkins, 2002), salinity (Latrach et al., 2014) and the presence of heavy metals (Klimek-kopyra et al., 2015), singly or in combination, could affect nodule development, legume plants growth and finally plant biomass. Thus, the effects of Rhizobium on legume plant growth promotion under high soil metal contamination are not always satisfactory, and remediation efficiency is still relatively low (Klimek-kopyra et al., 2015; Sanchezpardo, Fernandezpascual & Zornoza, 2012). An alternative to single inoculation of Rhizobium for enhancing plant growth has been to use mixed inoculation or coinoculation to improve plant growth (Kumar et al., 2017; Haro et al., 2018). A large number of such studies have reported that coinoculation could promote plant growth and increase metal accumulation capacity in plant tissue. Coinoculation of *Acinetobacter* sp. RG30 and *Pseudomonas putida* GN04 with phosphorus-soluble, IAA-producing and siderophore-producing bacteria significantly increased Cu extraction by maize (Rojas-Tapias, Bonilla & Dussán, 2014). Most of these studies were conducted for single heavy metals and low concentrations of heavy metals in soil. In fact, metal-contaminated soils are mostly caused by more than two kinds of heavy metals, and some heavy metal concentrations are very high in the soil. The ability of Rhizobium to convert nitrogen into ammonia is relevant for plant nutrition since nitrogen is an essential and sometimes limiting nutrient for plant growth in heavy metal contaminated soil. We therefore selected two bacterial strains with complementary functions (N_2_ fixing and Cu-resistant *Sinorhizobium meliloti* CCNWSX0020 and indole-producing and Zn-resistant *Agrobacterium tumefaciens* CCNWGS0286) as experimental subjects. The aims of this study were to (1) study the effects of coinoculated bacteria on the metal tolerance of *Medicago lupulina* under high concentrations of Cu or Zn stress; (2) study the effects of coinoculation on *Medicago lupulina* growth and Cu/Zn uptake under dual stress of high concentrations of Cu and Zn; and (3) by measuring the activity of antioxidant enzymes, identify the possible mechanism of coinoculation of *S. meliloti* and *A. tumefaciens* in alleviating Cu and Zn stress in plants. The results provide some insight into how coinoculation affected the antioxidant activity of host plants and enhanced legume defense systems to excess Cu/Zn, thus providing an efficient strategy to facilitate the ability of host plants to remediate heavy metal-contaminated soil.

## Material and Methods

### Bacterial growth conditions

All bacteria used in this study were listed in Table 1. The *Sinorhizobium meliloti* strain CCNWSX0020, Cu^2+^ resistance up to 1.6 mM in YMA medium, was isolated from the root nodules of *M. lupulina* growing in mine tailings in China (Fan et al., 2011). The draft genome of this strain was sequenced and annotated (GenBank accession number AGVV00000000), and genes related to copper resistance were predicted in previous studies (Li et al., 2012; Li et al., 2014). *Agrobacterium tumefaciens* CCNWGS0286 was isolated from the nodules of *Robinia pseudoacacia*, which grew in lead-zinc mine tailings in Gansu Province in northwestern China (Hao et al., 2012). *S. meliloti* CCNWSX0020 and *A. tumefaciens* CCNWGS0286 were grown at 28 °C with shaking at 180 rpm in tryptone yeast extract (TY) medium (5 g tryptone, 3 g yeast extract, and 0.7 g CaCl_2_, and 15 g agar per liter). The growth medium for the bacterial strains was supplemented with ampicillin (100 µg mL^−^^1^) when necessary.

**Table 1:** Bacteria used in the work.

Bacteria	Features	Source
**Strains**		
*S. meliloti* CNWSX0020	Wild type, Amp^r^	Reference (Fan et al., 2011)
*A. tumefaciens* CCNWGS0286	Wild type, Cm^r^,Km^r^	Reference (Hao et al., 2012)

### Plant growth conditions

To study the individual and combined effects of bacterial strains on plant growth and Cu^2+^/Zn^2+^ content in tissue, pot experiments were conducted with *M. lupulina*. Seeds of *M. lupulina* were surface sterilized by treatment with 95% ethanol for 1 min and 5% sodium hypochlorite for 3 min, and thoroughly rinsed with sterile distilled water several times. Sterilized seeds were germinated in petri dishes with water agar at 28 °C for 48 h. Germinated seedlings were sown in plastic planting bags (15 cm ×  30 cm) filled with 100 g sterilized mixture of vermiculite and perlite (3:2, v/v). The bags were divided into four groups. The first group was supplied with 0, 100, 200, and 400 mg/kg CuSO_4_ (the concentration of Cu applied was based on previous experiments) (Kong et al., 2015), the second group was supplied with 0, 100, 200, and 400 mg/kg ZnSO_4_, the third group contained the mixture of metallic compounds with a final concentration of 400 mg/kg CuSO_4_ and 400 mg/kg ZnSO_4_ and the fourth group did not supplement CuSO_4_ and ZnSO_4_. Seven seedlings were planted in each bag, and at least three replicates were conducted for each treatment. When the first main leaf grew out, suspensions of strain CCNWSX0020 (OD_600_ = 1.0), CCNWFS0286 (OD_600_ = 1.0) or the mixture of these two strains (1:1, V/V) were added to *M. lupulina* root with a final concentration of 10^8^ CFU per root. Seedlings without inoculation were included as blank controls. Fahraeus nitrogen-free nutrient solution (Fahraeus, 1957) was used for growing plants in a greenhouse with a 16/8 h photoperiod (light/dark) and at 25 ± 1 °C for 40 days. The plants were watered with 300 ml Fahraeus nutrient solution every seven days and 200 ml water every three days during the experiment.

### Plant growth, antioxidant enzyme and nitrogenase activity

Plants were harvested after 40 days, and the dry weight, shoot and root length, and number of nodules were determined and recorded. Nitrogenase activity in nodules was measured by the acetylene reduction assay as described by Hardy et al. (1968), with minor modifications. Nodules were taken from roots at 30 dpi (days post-inoculation). Samples (0.2 g) were placed into 100 ml glass vials and sealed with a rubber stopper, and then 1 ml air was vented with a syringe. After injection of 1 ml acetylene, the vials were incubated for 12 h at 28 °C. Acetylene and ethylene were measured with a Shimadzu GC-17A gas chromatograph (H-flame ionization detector, helium carrier gas, flow rate: 6 ml/min, column length: 30 m, inner diameter: 0.53 mm). The ethylene production by each sample was standardized using a standard ethylene curve. Plants were harvested at different developmental stages. The shoots and roots of all samples were detached and weighed. The shoots or roots samples (0.2 g) were homogenized in 5 ml of ice cold 50 mM potassium phosphate buffer (pH 7.8). The homogenate was then centrifuged at 12,000 ×g for 15 min at 4 °C, and the supernatant was used as enzyme extract. The catalase (CAT) activity was assayed by measuring of the decrease of H_2_O_2_ at a wavelength of 240 nm according to the method of Aebi (1984). Ascorbate peroxidase (APX) activity was determined according to the method of Nakano & Asada (1981). Superoxide dismutase (SOD) activity was measured by assaying the enzyme’s ability to inhibit the photochemical reduction of nitroblue tetrazolium (NBT) as described previously by Beauchamp & Fridovich (1971).

### Zn and Cu contents

The harvested plants were rinsed three times with sterilized deionized distilled water to remove any loosely bound Zn^2+^ or Cu^2+^, and then the above-ground portions and roots of *M. lupulina* were separated and dried at 80  °C. The 0.2000 g dry plant tissue samples were ground and digested with a nitric acid-perchloric acid mixture (HNO_3_ and HClO_4_ in a 5:1 ratio). The digestive solution was diluted to the required analytical range for zinc or copper determination. The copper or zinc concentration was analyzed with atomic absorption spectrophotometry (AAS, ZEEnit700P, Analytik Jena AG, Germany) using an external standard method. Total copper or zinc in plant tissues was calculated as follows:

Total Cu or Zn uptake = metal concentration × dry weight of plant tissues,

Metal concentration represents copper or zinc concentration in plant tissues measured by atomic absorption spectrophotometry, and dry weight of plant tissues represents the dry weight of the root or aboveground portion of each plant.

### Statistical analysis

All experiments in the present study were performed in at least three replicates. The data were analyzed by one-way analysis of variance (ANOVA). The significant differences between means were compared using Fisher’s protected LSD test at *P* ≤ 0.05. Statistical analysis was performed using SPSS software version 16.0 (Armonk, NY, USA).

## Results

### Effect of single or coinoculation on plant growth and dry weight under Cu or Zn stress

**Figure 1:**
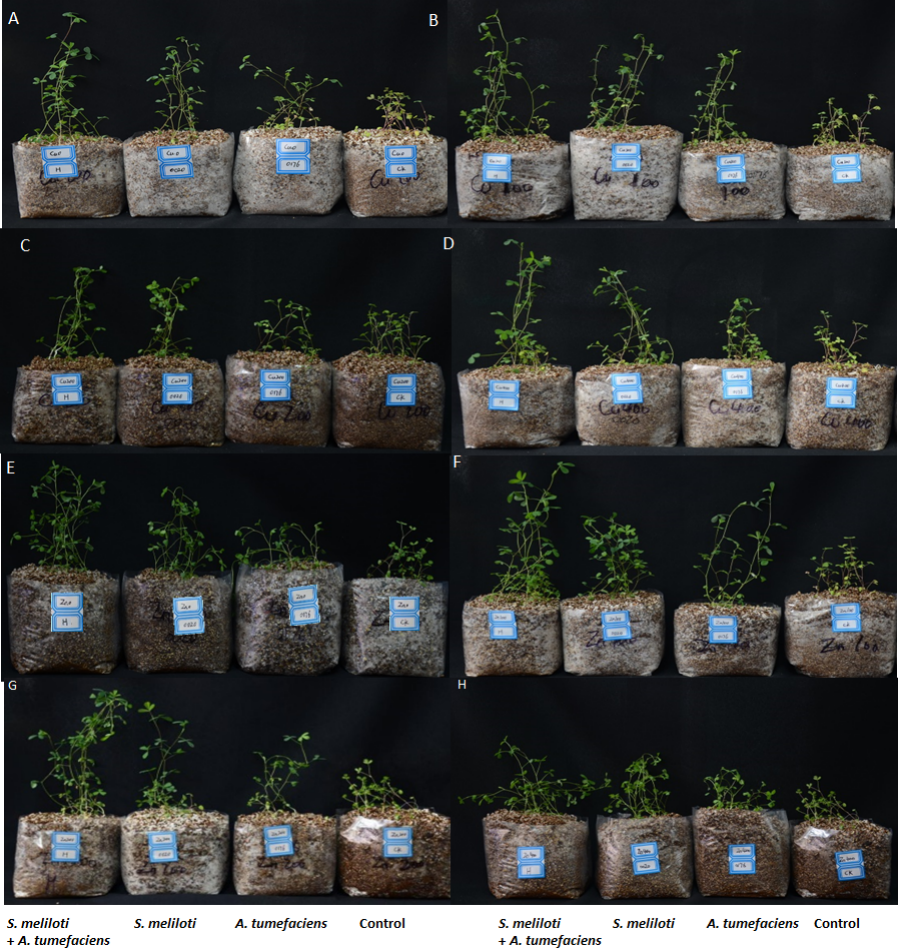
The growth of *M. lupulina* inoculated with *S. meliloti* and *A. tumefaciens* alone or combination in a pot with or without different concentrations of Cu/Zn and at 40 days after inoculation. (A) 0 mg kg^−1^, (B) 100 mg kg^−1^ CuSO_4_, (C) 200 mg kg^−1^ CuSO_4_, (D) 400 mg kg^−1^ CuSO_4_, (E) 0 mg kg^−1^ ZnSO_4_, (F) 100 mg kg^−1^ ZnSO_4_, (G) 200 mg kg^−1^ ZnSO_4_, (H) 400 mg kg^−1^ ZnSO_4_.

**Figure 2:**
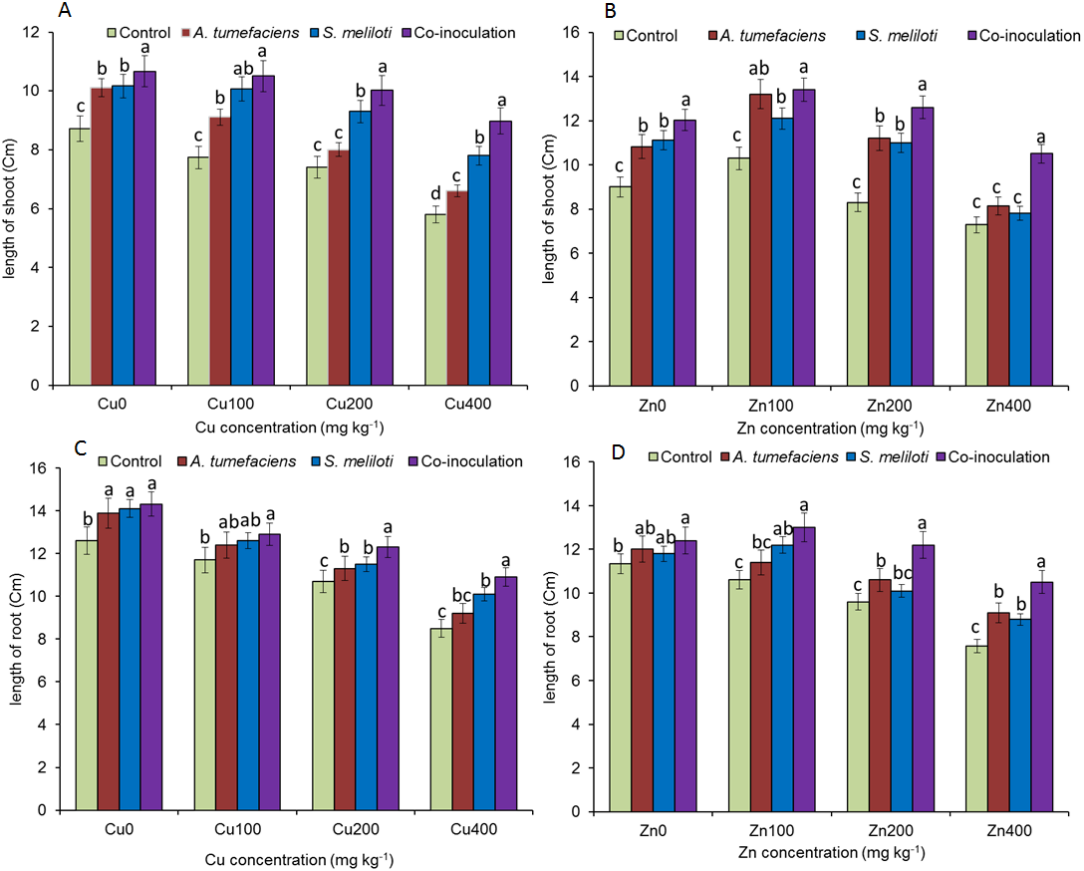
Effects of single or dual inoculation with *S. meliloti* and *A. tumefaciens* on root/aboveground portion length in the presence of Cu/Zn. Effects of single or dual inoculation with *S. meliloti* and *A. tumefaciens* on root/aboveground portion length in the presence of Cu/Zn. Aboveground portion length (AB); root length (CD). Values are means ± SE. abcd letters on the bars denote differences on the basis of a *t*-test (*p* < 0.05).

**Figure 3:**
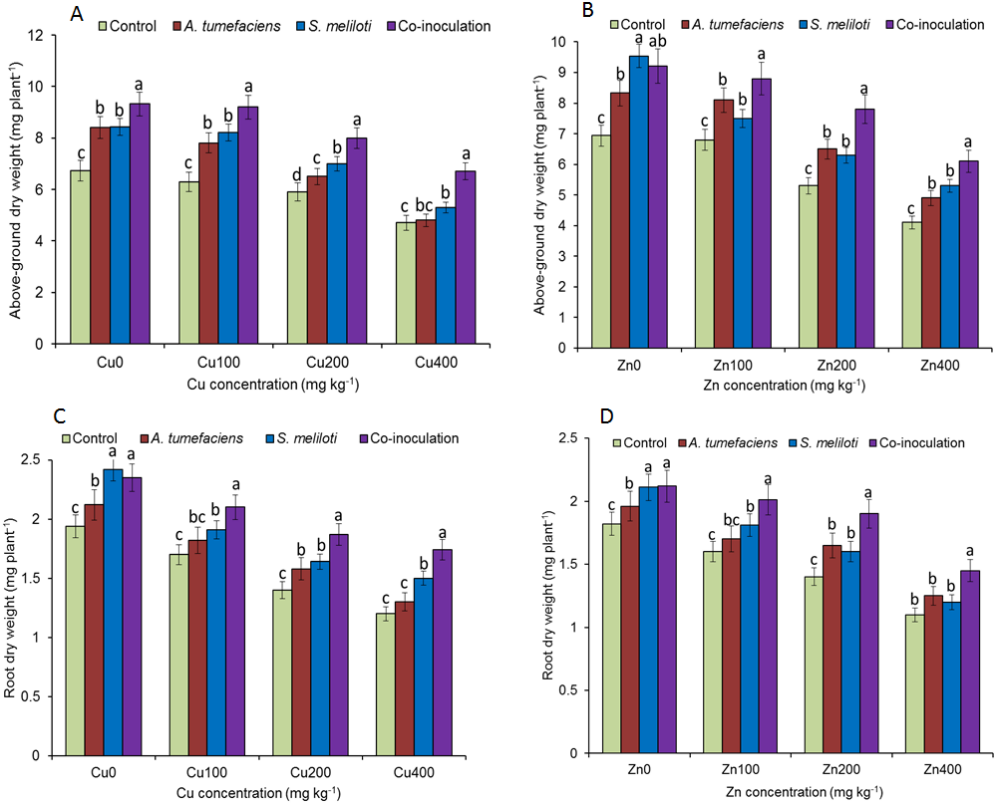
Effects of single or dual inoculation with *S. meliloti* and *A. tumefaciens* on root/aboveground portion dry weight in the presence of Cu/Zn. Aboveground portion dry weight (AB); root dry weight (CD). Values are means ± SE. abc letters on the bars denote differences on the basis of a *t*-test (*p* < 0.05).

Analysis of the effects of different CuSO_4_ levels on shoot length, root length, and dry weight of the host plant was performed. With increasing copper concentration, the growth and biomass of *M. lupulina* were gradually inhibited. When the concentration of CuSO_4_ reached 400 mg kg^−1^, the leaves of plants appeared to be yellowing and falling (Fig. 1). However, application of rhizobacteria alone or in combination significantly (*P* ≤ 0.05) increased the growth of *M. lupulina* compared to that of uninoculated controls. Under 400 mg kg^−1^ Cu^2+^ treatment conditions, single inoculation of *S. meliloti* or *A. tumefaciens* showed somewhat increased plant length and biomass. Compared with the uninoculated treatment, the shoot length of *M. lupulina* inoculated with *S. meliloti* or *A. tumefaciens* was increased by 29.6% or 13.3%, respectively. The root length of *Sinorhizobium*-inoculated or *Agrobacterium*-inoculated plants was 16.3% or 7.7% longer, respectively, than the control. The dry weight of the roots was increased up to 24.8% and 9.9%, respectively, over that of the control. Coinoculation of *S. meliloti* and *A. tumefaciens* significantly increased the shoot length, root length, root dry weight and above-ground protion dry weight of the host plant by 53.9%, 29.2%, 55.4% and 52.9%, respectively, compared to those of the control (Fig. 2 and Fig. 3).

The increases in plant length and dry weight also displayed the same trend when inoculated with *S. meliloti*, *A. tumefaciens* or a combination of these two bacteria under 400 mg kg^−1^ Zn^2+^ stress. Inoculation with *S. meliloti*, *A. tumefaciens* or *S. meliloti* + *A. tumefaciens* increased root length by 16.0%, 19.9% or 38.5% (Shoot length enhanced by 7.0%, 11.7% or 44.2%) in the presence of 400 mg kg^−1^ Zn^2+^. The shoot and root dry weights of *M. lupulina* inoculated with *S. meliloti*, *A. tumefaciens* and *S. meliloti* + *A. tumefaciens* were 35.0%, 27.8%, 64.7% and 9.0%, 14.9%, 34.6%, respectively, more than those of uninoculated plant (Figs. 2 and 3).

### Effect of single or coinoculation on plant growth under combination stress of copper and zinc

**Figure 4:**
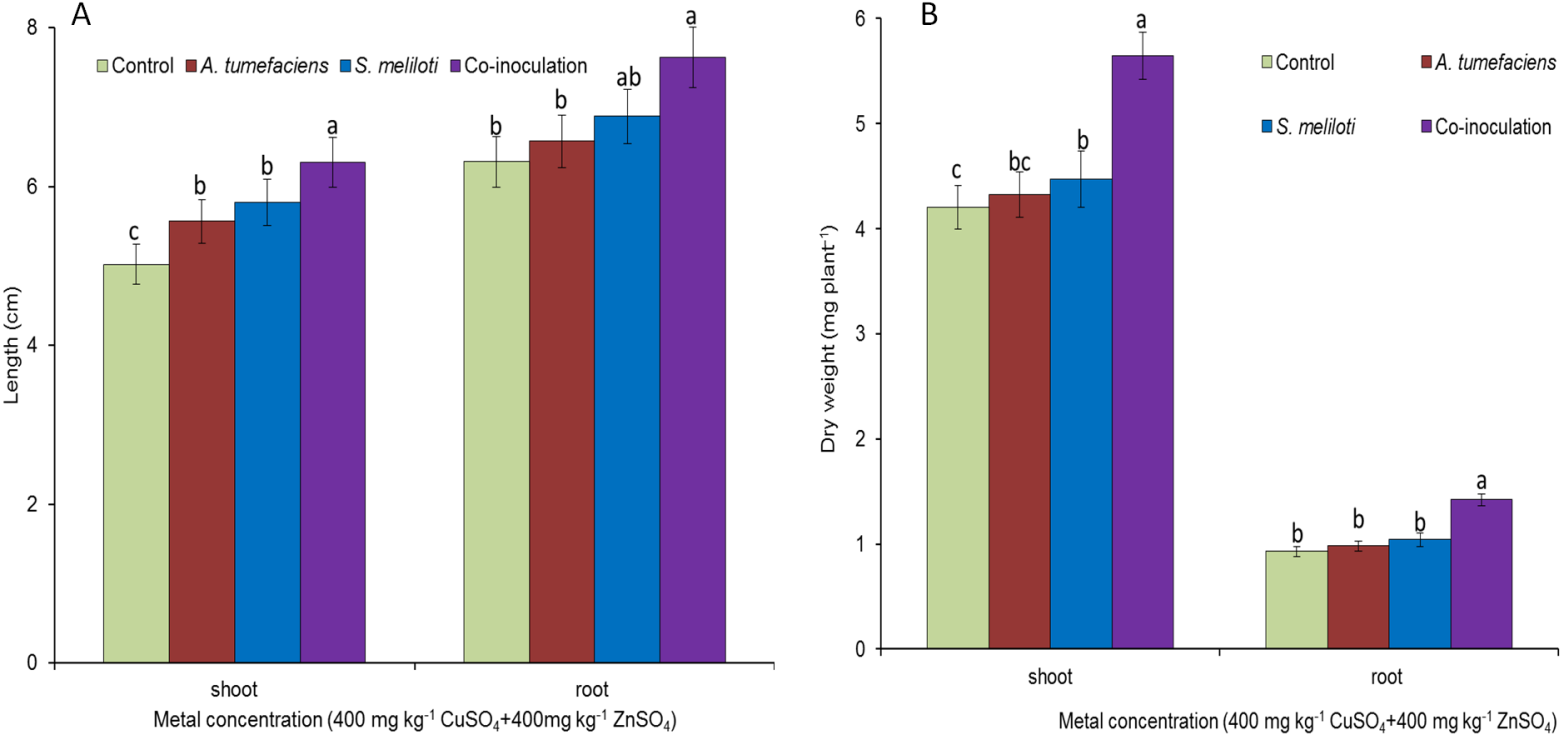
Effects of single or dual inoculation with *S. meliloti* and *A. tumefaciens* on root/aboveground portion length and dry weight under double stress of Cu and Zn. Aboveground plant and root length (A); aboveground plant and root dry weight (B). Values are means ± SE. abc letters on the bars denote differences on the basis of a *t*-test (*p* < 0.05).

To determine the effect of rhizobacteria on plant growth under multiple-metal stress, *M. lupulina* seedlings were inoculated with a single species of bacteria or a combination of *S. meliloti* and *A. tumefaciens* in the presence of 400 mg kg^−1^ CuSO_4_ and 400 mg kg^−1^ ZnSO_4_. High-concentration copper and zinc mixed stress was more toxic to plants, and plant growth was seriously affected. To a certain extent, single inoculation could promote the growth of plants. However single inoculation increased the dry weight and length of *M. lupulina* to a relatively small extent. Root and aboveground portion length increased up to 9.0% and 16.6% over those of the control for *S. meliloti* (5.3% and 12.1% for *A. tumefaciens*), repectively, and root and aboveground portion dry weight increased up to 6.6% and 12.9% over those of the control for *S. meliloti* (2.8% and 5.1% for *A. tumefaciens*), respectively (Fig. 4). However, dual inoculation significantly increased plant growth. The length and dry weights of the above-ground protion were 32.6% and 34.3% greater, respectively, than those of control, and the increases in *M. lupulina* root length (up to 14.1% over that of the control) and dry weight (up to 31.2% over that of the control) also displayed the same trend under double stress of 400 mg kg^−1^ copper and zinc (Fig. 4).

### Cu^2+^ and Zn^2+^ contents in the tissue of *M. lupulina* inoculated with *S. meliloti* and *A. tumefaciens*

The copper and zinc contents in both aboveground portions and roots of plants dual inoculated with *S. meliloti* and *A. tumefaciens* were elevated when the Cu^2+^ and Zn^2+^ concentrations were 400 mg kg^−1^ in the medium, an effect that was more pronounced in roots than in aerial plant tissues. There were no significant changes in heavy Cu and Zn contents in aboveground tissues per unit weight between dual-inoculation and single-inoculation plants in the presence of 400 mg kg^−1^ Cu^2+^ and Zn^2+^. Furthermore, a significant increase was observed when comparing the Cu and Zn contents in the roots of dual-inoculated plants with those of single-inoculated plants or control plants in the presence of 400 mg kg^−1^ Cu^2+^ and Zn^2+^. The total amount of Cu/Zn accumulated in each plant was calculated by multiplying the Cu/Zn content by plant tissue dry weight. The results showed that the total Cu and Zn uptake in each dual-inoculated plant was significantly greater than those in the plants inoculated with a single bacterial strain or in control plants under the double stress of high concentrations of copper and zinc. Compared with *S. meliloti* or *A. tumefaciens*, coinoculation increased total Cu uptake in *M. lupulina* by 39.1% and 47.5% and increased total Zn uptake by 35.4% and 44.2% in the presence of 400 mg kg^−1^ Cu^2+^ and Zn^2+^ (Table 2).

**Table 2:** Uptake of heavy metal in *M. lupulina*.

Treatment	Cu400	Cu400+Zn400	Zn400	Cu400+Zn400
	Copper uptake		Zinc uptake	
Non-inoculation	2.98 ± 0.18b	2.01 ± 0.15b	3.59 ± 0.13c	2.51 ± 0.43b
*A. tumefaciens* CCNWGS0286	3.68 ± 0.21ab	2.17 ± 0.18b	4.22 ± 0.15b	3.21 ± 0.14b
*S. meliloti* CNWSX0020	4.34 ± 0.32a	2.30 ± 0.20b	4.06 ± 0.21b	3.42 ± 0.18b
*S. meliloti* CNWSX0020 + *A. tumefaciens* CCNWGS0286	4.73 ± 0.31a	3.20 ± 0.21a	4.56 ± 0.31a	4.63 ± 0.22a

**Notes.**

Cu400 the final concentration of CuSO_4_ in plastic bag was 400 mg kg^−1^ Zn400 the final concentration of ZnSO_4_ in plastic bag was 400 mg kg^−1^ Cu400+Zn400 the final concentration of CuSO_4_ and ZnSO_4_ in plastic bag was 400 mg kg^−1^, respectively

The values indicate the mean ± SE of for replicates. Different letters (a and b) show significant difference between treatments at *p* <0.05 by Duncan test.

### Effect of dual inoculation or single inoculation on nodulation

**Figure 5:**
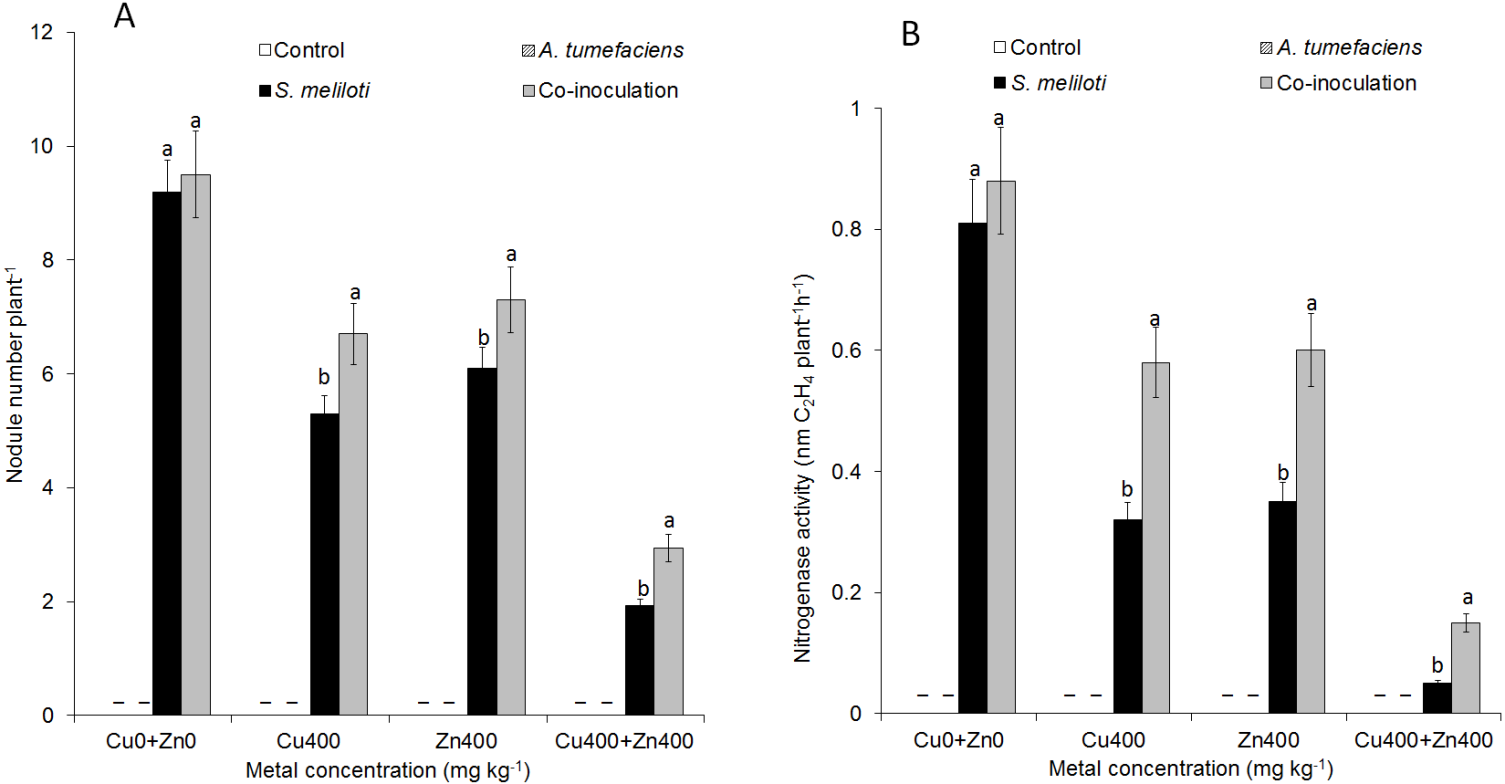
Nodule number and nitrogenase activity. Nodule number (A) and nitrogenase activity (B) of *M. lupulina* inoculated *S. meliloti* or a combination of *S. meliloti* and *A. tumefaciens* under moderate (200 mg kg^−1^) or severe (400 mg kg^−1^) Cu or Cu (400 mg kg^−1^) and Zn (400 mg kg^−1^) double stress conditions. –No nodule was observed. The values indicate the means ± SE of three replicates. Bars carrying different letters denote differences on the basis of a *t*-test (*p* < 0.05).

For selected concentrations of Cu or Zn, all the plants inoculated with *S. meliloti* could form nodules. However, the nodulation efficiency was obviously different. There was no difference in nodule number between dual inoculation and single inoculation of *S. meliloti* without Cu^2+^. The nodule number and nitrogenase activity of the dual inoculation plants were 35.8% and 87.5% greater than *S. meliloti*-inoculated plants under 400 mg kg^−1^ Cu^2+^ stress (Fig. 5). Although nodule number and nitrogenase activity decreased significantly under the double stress of 400 mg kg^−1^ Cu and Zn, the nodule number and nitrogenase of dual inoculation plants were still 48.5% and 154.4% higher, respectively, than those of the plants inoculated with *S. meliloti* alone (Fig. 5). The N content in aerial parts of the dual inoculation plants was significantly higher than that of the single inoculation or control plants in all treatments. However, the N content in roots of dual inoculation plants was not significantly changed by treatment with the double stress of 400 mg kg^−1^ Cu^2+^ and Zn^2+^ compared to that of other treatments (Fig. S1).

### Effect of dual inoculation or single inoculation on plant antioxidant enzyme activity

**Figure 6:**
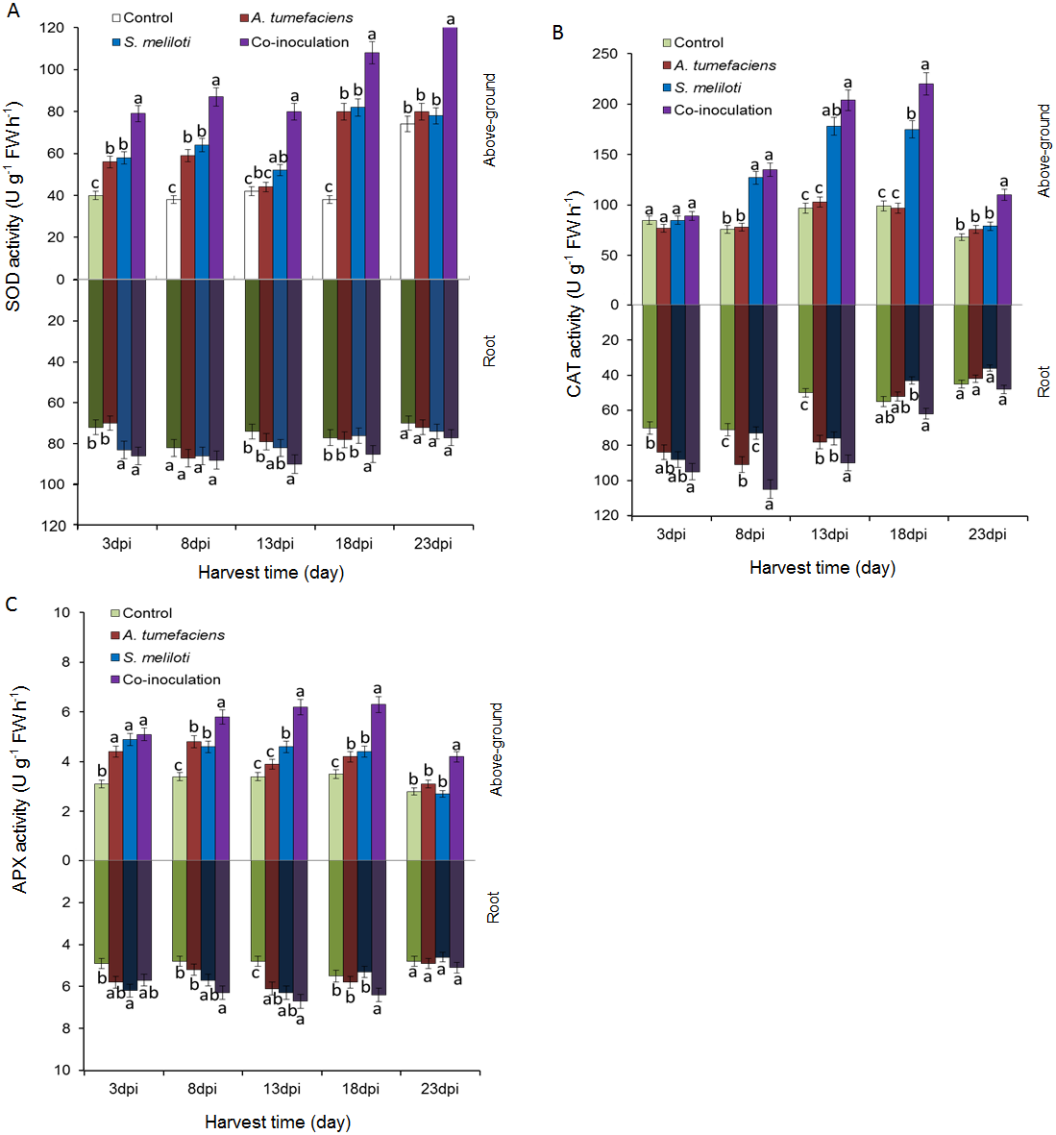
Effects of single or dual inoculation with *S. meliloti* and *A. tumefaciens* on SOD (A), CAT (B), APX (C) activities in shoots (white bar) and roots (gray bar) of *M. lupulina* under Cu (400 mg kg^−1^) and Zn (400 mg kg^−1^) double stress conditions. The values indicate the means ± SE of three replicates. Bars carrying different letters denote differences on the basis of a *t*-test (*p* < 0.05).

To determine whether the antioxidant enzyme activities of plants are enhanced by the inoculation of *S. meliloti* and *A. tumefaciens* alone or in combination, we analyzed SOD, CAT and APX in aboveground portion and root of *M. lupulina* under 400 mg kg^−1^ Cu^2+^ and Zn ^2+^ stress. The antioxidant enzyme activities were significantly increased by the rhizobacteria inoculation of *M. lupulina* compared to those of the uninoculated control. As shown in Fig. 6A, the total SOD activity in the aboveground portion of dual-inoculated plants increased by 36.8%, 37.1%, 52.9%, 32.1% and 64.6% at 3, 8, 13, 18 and 23 dpi, respectively, compared with that of *S. meliloti*-inoculated plants in the presence of Cu and Zn (up to 41.8%, 47.7%, 81.4%, 35.4% and 60.5%, respectively, over that of *A. tumefaciens*-inoculated plants). No significant increase was observed in roots for any inoculation treatment.

Figure 6B showes that the CAT activities in the aerial part of dual inoculation plants were higher than those of the *A. tumefaciens* inoculation plants in the presence of Cu and Zn from 8 to 23 dpi, and a significant increase was observed in the aerial part of the dual inoculation plants in comparison to the *S. meliloti* inoculation plants at 18 and 23 dpi. The CAT activities in roots of dual inoculation plants were significantly elevated only at 8 and 18 dpi.

As shown in Fig. 6C, compared with single inoculation, the increase in APX activities in roots of dual inoculation plants was not obvious. However, the APX activities in the shoots of dual inoculation plants were elevated in comparison to those of each single inoculation plant from 8 to 23 dpi.

## Discussion

Many kinds of transition metals, such as copper and zinc, are essential elements for plant growth. These transition metals are involved in a wide variety of metabolic pathways at low concentrations. However, they are toxic to plants if their concentrations exceed normal levels (Seregin & Kozhevnikova, 2006). In this study, seedling growth was significantly inhibited when 400 mg kg^−1^ Cu^2+^ and Zn^2+^ were applied (Figs. 1 and 2). The results were consistent with those for lead and cadmium, which decreased root length, shoot length and percent germination (Hassan et al., 2016). The decline in *M. lupulina* biomass was presumably due to root damage or oxidative stress caused by excess Cu and Zn (Liu et al., 2018). The adverse effects reduced the uptake of essential mineral nutrients, altered water balance, and decreased the activity of various enzymes and chlorophyll (Muradoglu et al., 2015; Alyemeni et al., 2018; Li et al., 2015).

Plants maintain many complex relationships with diverse soil organisms, such as bacteria, protozoa, fungi, nematodes and annelids, living around them (Gange, Eschen & Schroeder, 2012). When seeds germinate and plants grow, they typically acquire specific bacteria and fungi that exist in the native soil. The influence of soil microorganisms on soil quality and plant health has recently received more emphasis. These microorganisms can promote plant acquisition of nutrients (Vimal et al., 2017), mineralization of organic phosphorus (Meyer et al., 2017), and production of phytohormones (Kurepin et al., 2015), alleviating the negative effects of environmental stress. Some metal-resistant microorganisms could also promote plant growth under heavy metal stress conditions; thus, more plant biomass increases the efficiency of phytoremediation. Dabrowska et al. (2017) observed that bacterial strains could promote plant growth by *Brassica napus* L. under Cd, Cu, Pb and Zn stresses. Therefore, plant growth-promoting bacteria (PGPB) have been widely used to increase the capacity of host plants to tolerate and absorb heavy metals from soil (Kamran et al., 2017). In the current study, all treatments of PGPB either alone or in combination showed significant positive influences on plant growth under Cu and Zn stress (Fig. 4). Although either single or dual inoculations of *M. lupulina* with the bacteria also increased the dry weight and length of plant roots and aboveground portions compared with those of uninoculated controls, dual inoculation of *S. meliloti* and *A. tumefaciens* produced significantly more dry weight than single inoculation of any bacteria (Figs. 3 and 4). PGPB are able to promote plant growth through one or several mechanisms, such as the production of phytohormones such as indole-3-acetic acid (IAA), siderophores, ACC-deaminase and phosphate solubilization (Yu et al., 2017; Chandra et al., 2018). PGPB also enhance symbiotic nitrogen fixation through promoting root development in general and root hair formation in particular, resulting in more potential colonization sites for rhizobia (Ahemad & Kibret, 2014). Based on the analysis of the features of *A. tumefaciens* CCNWGS0286 in previous work, this bacterium could produce high levels of IAA even under 1.0 mM Zn stress (Hao et al., 2012). Although other plant growth-promoting traits, such as acetoin production, ACC deaminase activity, and organic P solubilization abilities were not detected, *A. tumefaciens* CCNWGS0286 could still promote the growth of *Robinia pseudoacacia* under heavy metal stress (Hao et al., 2012). Additionally, *S. meliloti* CCNWSX0020 can establish a normal symbiotic relationship with the host plant under Cu stress since it is tolerant to excess Cu, and nitrogenase activity could be detected in this study, indicating that effective nodules were formed under Cu and Zn stress (Fig. 5). However, the N content in *M. lupulina* showed no significant increase compared with that in control plants under Cu and Zn stress (Fig. S1). This might be because the negative effects of heavy metals reduced the number of nodules and decreased nitrogenase activity.

The positive effects of coinoculation of *M. lupulina* with *S. meliloti* and *A. tumefaciens* were also observed. A significant increase in N content in plants was detected after coinoculation of *S. meliloti* and *A. tumefaciens,* which significantly increased the N content of the above-ground portion by 19.1% compared to that of control in the presence of 400 mg kg^−1^ Cu^2+^ and Zn^2+^. Similar results were observed by Sepulvedacaamano et al. (2018), who found that coinoculation of lentil with *Rhizobium leguminosarum bv. viciae* AG-84 and *Pseudomonas umsongensis* LY50a improved the progression of nodulation by 85.0% and increased nitrogen fixation in comparison to those of rhizobia inoculation alone. Coinoculation leading to high biomass might be attributed to IAA produced by plant growth-promoting bacteria. IAA increases plant cell division, enlarges the root system and the number of Rhizobium-infection sites (Tanimoto, 2005), and subsequently promotes nitrogen uptake and plant growth.

To analyze effects on heavy metal accumulations in *M. lupulina* of single or synergistic inoculation with *S. meliloti* and *A. tumefaciens* under Cu/Zn stress, the total metal uptake in plant tissues was measured. The results showed that inoculation with either *A. tumefaciens* or *S. meliloti* significantly increased the total uptake of Cu or Zn in plants under single metal stress (Table 2). This result might be due to the fact that the bacteria we used could promote plant growth by producing plant growth-regulating substances, N fixation or effects on metal solubility and bioavailability, all of which affect metal uptake (Pajuelo et al., 2011). However, under double metal stresse conditions, the total metal absorbed by the plants under different inoculation treatments was less than that under single metal stress, except for Zn uptake by plants inoculated with *A. tumefaciens* and *S.  meliloti*. Our results also indicated that there was no significant difference in the amount of heavy metals absorbed by noninoculated and single-inoculated plants under 400 mg kg^−1^ Cu and Zn stress. Compared with *S. meliloti* and *A. tumefaciens* single inoculation, coinoculation increased total Cu uptake by 39.1% and 47.5% and increased total Zn uptake by 35.4% and 44.2%, respectively (Table 2). We speculated that the growth of single bacterium was inhibited under the combined stress of two high concentrations of heavy metals, thereby reducing beneficial effects on plants. Metal-resistant complementary bacteria (*S. meliloti* tolerant to Cu and *A. tumefaciens* tolerant to Zn) could survive and increase nutrition, plant biomass and tolerance of plants to Cu/Zn stress (Fatnassi et al., 2015). Excessive heavy metals not only hamper metabolic processes in plant cells, but also increase the generation of reactive oxygen species (ROS) such as superoxide free radicals (O_2_^−^), hydroxyl free radicals (OH^−^), and hydrogen peroxide (H_2_O_2_) through Fenton-like reactions (Tsang et al., 1996). The elevated levels of ROS can cause oxidative stress via disturbing the redox status equilibrium in plant cells. Usually, superoxide dismutase (SOD), catalase (CAT), ascorbate peroxidase (APX), and glutathione reductase (GR) are protective enzymes of the glutathione (GSH) peroxidase system to handle external oxidative stress, which can directly or indirectly clear intracellular excessive ROS. Becana et al. (2000) suggested that the promotion of plant antioxidant defenses by rhizobia may improve symbiotic performance, especially under nonoptimal conditions. Kong et al. (2015) showed that the expression level of antioxidant genes, i.e., CuZnSODc, CuZnSODp, CAT, and APXc, in plants increased in the presence of excess Cu(II) when the plants were inoculated with Rhizobium. We detected the activity of CAT, APX and SOD in different plant tissues and found that the enzyme activities of the coinoculated plants were enhanced in comparison with those of noninoculated or single-inoculated plants at different growth stages under Cu/Zn stress (Fig. 6). In addition, *S. meliloti* could produce acidic exopolysaccharides, which act as a diffusion barrier against H_2_O_2_ (Davies & Walker, 2007). Thus, it is speculated that coinoculation leads to increased nitrogen nutrition and antioxidant enzyme activities, thereby alleviating heavy metal toxicity and enhancing metal ion accumulation in plant tissue.

## Conclusion

Excessive heavy metal decreases nodulation and reduces nitrogenase activity of *M. lupulina*. The coinoculation of host plants with *S. meliloti* and PGPB *A. tumefaciens* alleviated heavy metal toxicity and promoted plant growth. These two strains have one or more properties of nitrogen fixation, copper and zinc resistance and IAA production. It is likely that coinoculation improved plant growth via these properties. When grown in medium containing high concentrations of Cu and Zn, we found that the roots of the coinoculated plants accumulated more Cu and Zn. The results confirm the importance of coinoculation to improve the heavy metal tolerance of plants and promote plant growth and phytostabilization efficiency.

##  Supplemental Information

10.7717/peerj.6875/supp-1 Figure S1 Nitrogen content of the aboveground portion and root of *M. lupulina* inoculated with *S. meliloti* or a combination of *S. meliloti* and *A. tumefaciens.* Nitrogen content of the aboveground portion (A) and root (B) of *M. lupulina* inoculated with *S. meliloti* or a combination of *S. meliloti* and *A. tumefaciens* under moderate (200 mg kg^−1^) or severe (400 mg kg^−1^) Cu or Cu (400 mg kg^−1^) and Zn (400 mg kg^−1^) double stress conditions. The values indicate the means ± SE of three replicates. Bars carrying different letters denote differences on the basis of a *t*-test (*p* < 0.05).

## Additional Information and Declarations

### Data Availability

The following information was supplied regarding data availability: The raw data is available at NCBI, accession number: AGVV00000000.

## References

[ref-1] Aebi H (1984). Catalase in vitro. Methods in Enzymology.

[ref-2] Ahemad M, Kibret M (2014). Mechanisms and applications of plant growth promoting rhizobacteria: current perspective. Journal of King Saud University - Science.

[ref-3] Ajina T, Sallem A, Haouas Z, Mehdi M (2017). Total antioxidant status and lipid peroxidation with and without in vitro zinc supplementation in infertile men. Andrologia.

[ref-4] Ali B, Amna Javed MT, Ali H, Munis MF, Chaudhary HJ (2017). Influence of endophytic *Bacillus pumilus* and EDTA on the phytoextraction of Cu from soil by using *Cicer arietinum*. International Journal of Phytoremediation.

[ref-5] Alyemeni MN, Ahanger MA, Wijaya L, Alam P, Bhardwaj R, Ahmad P (2018). Selenium mitigates cadmium-induced oxidative stress in tomato (*Solanum lycopersicum* L.) plants by modulating chlorophyll fluorescence, osmolyte accumulation, and antioxidant system. Protoplasma.

[ref-6] Ampofo EA, Awortwe D (2017). Heavy metal (Cu, Fe and Zn) pollution in soils: pig waste contribution in the central region of Ghana. Advances in Applied Science Research.

[ref-7] Bano A, Hussain J, Akbar A, Mehmood K, Anwar M, Hasni MS, Ullah S, Sajid S, Ali I (2018). Biosorption of heavy metals by obligate halophilic fungi. Chemosphere.

[ref-8] Barauna AC, Rouws LF, Simoesaraujo JL, Fb DR, Iannetta PP, Maluk M, Goi SR, Reis VM, James EK, Zilli JE (2016). *Rhizobium altiplani* sp. nov. isolated from effective nodules on *Mimosa pudica* growing in untypically alkaline soil in central Brazil. International Journal of Systematic and Evolutionary Microbiology.

[ref-9] Beauchamp C, Fridovich I (1971). Superoxide dismutase: improved assays and an assay applicable to acrylamide gels. Analytical Biochemistry.

[ref-10] Becana M, Dalton DA, Moran JF, Iturbe-Ormaetxe I, Matamoros MA, Rubio CM (2000). Reactive oxygen species and antioxidants in legume nodules. Physiologia Plantarum.

[ref-11] Chandra D, Srivastava R, Glick BR, Sharma AK (2018). Drought-tolerant *Pseudomonas* spp. improve the growth performance of finger millet (*Eleusine coracana* (L.) Gaertn.) under non-stressed and drought-stressed conditions. Pedosphere.

[ref-12] Dabrowska G, Hrynkiewicz K, Trejgell A, Baum C (2017). The effect of plant growth-promoting rhizobacteria on the phytoextraction of Cd and Zn by *Brassica napus* L. International Journal of Phytoremediation.

[ref-13] Davies BW, Walker GC (2007). Identification of novel *Sinorhizobium meliloti* mutants compromised for oxidative stress protection and symbiosis. Journal of Bacteriology.

[ref-14] Ebbs SD, Kochian LV (1997). Toxicity of zinc and copper to Brassica species: implications for phytoremediation. Journal of Environmental Quality.

[ref-15] Fahraeus G (1957). The infection of clover root hairs by nodule bacteria studied by a simple glass slide technique. Journal of General Microbiology.

[ref-16] Fan L, Ma Z, Liang J, Li H, Wang E, Wei G (2011). Characterization of a copper-resistant symbiotic bacterium isolated from *Medicago lupulina* growing in mine tailings. Bioresource Technology.

[ref-17] Fatima H, Ahmed A (2018). Micro-remediation of chromium contaminated soils. PeerJ.

[ref-18] Fatnassi IC, Chiboub M, Saadani O, Jebara M, Jebara SH (2015). Impact of dual inoculation with Rhizobium and PGPR on growth and antioxidant status of *Vicia faba* L. under copper stress. Comptes Rendus Biologies.

[ref-19] Gange AC, Eschen R, Schroeder V, Glenn RI, Dicke M, Hartley SE (2012). The soil microbial community and plant foliar defenses against insects. The ecology of plant secondary metabolites: from genes to global processes.

[ref-20] Hao X, Taghavi S, Xie P, Orbach MJ, Alwathnani HA, Rensing C, Wei G (2014). Phytoremediation of heavy and transition metals aided by legume-rhizobia symbiosis. International Journal of Phytoremediation.

[ref-21] Hao X, Xie P, Johnstone L, Miller SJ, Rensing C, Wei G (2012). Genome sequence and mutational analysis of Plant-Growth-Promoting bacterium *Agrobacterium tumefaciens* CCNWGS0286 isolated from a Zinc-Lead mine tailing. Applied and Environmental Microbiology.

[ref-22] Hardy RW, Holsten RD, Jackson EK, Burns RC (1968). The acetylene-ethylene assay for N_2_ fixation: laboratory and field evaluation. Plant Physiology.

[ref-23] Haro H, Sanon KB, Roux CL, Duponnois R, Traore AS (2018). Improvement of cowpea productivity by rhizobial and mycorrhizal inoculation in Burkina Faso. Symbiosis.

[ref-24] Hassan W, Bano R, Bashir S, Aslam P (2016). Cadmium toxicity and soil biological index under potato (*Solanum tuberosum* L.) cultivation. Soil Res.

[ref-25] Huang G, Su X, Rizwan M, Zhu Y, Hu H (2016). Chemical immobilization of Pb, Cu, and Cd by phosphate materials and calcium carbonate in contaminated soils. Environmental Science and Pollution Research.

[ref-26] Kamran M, Bibi S, Xu R, Hussain S, Mehmood K, Chaudhary HJ (2017). Phyto-extraction of chromium and influence of plant growth promoting bacteria to enhance plant growth. Journal of Geochemical Exploration.

[ref-27] Khadka N, Milton RD, Shaw S, Lukoyanov D, Dean DR, Minteer SD, Raugei S, Hoffman BM, Seefeldt LC (2017). Mechanism of nitrogenase H_2_ formation by metal-hydride protonation probed by mediated electrocatalysis and H/D isotope effects. Journal of the American Chemical Society.

[ref-28] Klimek-kopyra A, Baran A, Zajac T, Kulig B (2015). Effects of heavy metals from polluted soils on the roots and nodules formation. Bulgarian Journal of Agricultural Science.

[ref-29] Kong Z, Deng Z, Glick BR, Wei G, Chou M (2017). A nodule endophytic plant growth-promoting *Pseudomonas* and its effects on growth, nodulation and metal uptake in *Medicago lupulina* under copper stress. Annals of Microbiology.

[ref-30] Kong Z, Mohamad OA, Deng Z, Liu X, Glick BR, Wei G (2015). Rhizobial symbiosis effect on the growth, metal uptake, and antioxidant responses of Medicagolupulina under copper stress. Environmental Science and Pollution Research.

[ref-31] Kumar A, Maurya BR, Raghuwanshi R, Meena VS, Islam MT (2017). Co-inoculation with Enterobacter and Rhizobacteria on yield and nutrient uptake by wheat (*Triticum aestivum* L.) in the alluvial soil under Indo-Gangetic plain of India. Journal of Plant Growth Regulation.

[ref-32] Kuramshina ZM, Smirnova YV, Khairullin RM (2018). Cadmium and Nickel toxicity for *Sinapis alba* plants inoculated with endophytic strains of *Bacillus subtilis*. Russian Journal of Plant Physiology.

[ref-33] Kurepin LV, Park MJ, Lazarovits G, Bernards MA (2015). *Burkholderia phytofirmans*-induced shoot and root growth promotion is associated with endogenous changes in plant growth hormone levels. Plant Growth Regulation.

[ref-34] Latrach L, Farissi M, Mouradi M, Makoudi B (2014). Growth and nodulation of alfalfa-rhizobia symbiosis under salinity: electrolyte leakage, stomatal conductance, and chlorophyll fluorescence. Turkish Journal of Agriculture and Forestry.

[ref-35] Li S, Yang W, Yang T, Chen Y, Ni W (2015). Effects of cadmium stress on leaf chlorophyll fluorescence and photosynthesis of *Elsholtzia argyi*-A cadmium accumulating plant. International Journal of Phytoremediation.

[ref-36] Li ZF, Ma ZQ, Hao XL, Rensing C, Wei GH (2014). Genes conferring copper resistance in *Sinorhizobium meliloti* CCNWSX0020 also promote the growth of *Medicago lupulina* in copper-contaminated soil. Applied and Environmental Microbiology.

[ref-37] Li Z, Ma Z, Hao X, Wei G (2012). Draft genome sequence of *Sinorhizobium meliloti* CCNWSX0020, a nitrogen-fixing symbiont with copper tolerance capability isolated from lead-zinc mine tailings. Journal of Bacteriology.

[ref-38] Liu L, Li J, Yue F, Yan X, Wang F, Bloszies S, Wang Y (2018). Effects of arbuscular mycorrhizal inoculation and biochar amendment on maize growth, cadmium uptake and soil cadmium speciation in Cd-contaminated soil. Chemosphere.

[ref-39] Malinowska E, Jankowski K (2017). Copper and zinc concentrations of medicinal herbs and soil surrounding ponds on agricultural land. Landscape and Ecological Engineering.

[ref-40] Meyer G, Bunemann EK, Frossard E, Maurhofer M, Mader P, Oberson A (2017). Gross phosphorus fluxes in a calcareous soil inoculated with *Pseudomonas protegens* CHA0 revealed by 33P isotopic dilution. Soil Biology and Biochemistry.

[ref-41] Mleczek M, Golinski P, Krzeslowska M, Gąsecka M, Magdziak Z, Rutkowski P, Budzynska S, Waliszewska B, Kozubik T, Karolewski Z, Niedzielski P (2017). Phytoextraction of potentially toxic elements by six tree species growing on hazardous mining sludge. Environmental Science and Pollution Research.

[ref-42] Muradoglu F, Gundogdu M, Ercisli S, Encu T, Balta F, Jaafar HZ, Ziaulhaq M (2015). Cadmium toxicity affects chlorophyll a and b content, antioxidant enzyme activities and mineral nutrient accumulation in strawberry. Biological Research.

[ref-43] Nakano Y, Asada K (1981). Hydrogen peroxide is scavenged by ascorbate-specific peroxidase in spinach chloroplasts. Plant and Cell Physiology.

[ref-44] Pajuelo E, Rodríguez-Llorente ID, Lafuente A, Caviedes MÁ (2011). Legume—*Rhizobium* symbioses as a tool for bioremediation of heavy metal polluted soils. Biomanagement of metal-contaminated soils.

[ref-45] Paungfoolonhienne C, Lonhienne TG, Yeoh YK, Webb RI, Lakshmanan P, Chan CX, Lim PE, Ragan MA, Schmidt S, Hugenholtz P (2014). A new species of Burkholderia isolated from sugarcane roots promotes plant growth. Microbial Biotechnology.

[ref-46] Peltzer S, Abbott LK, Atkins CA (2002). Effect of low root-zone temperature on nodule initiation in narrow-leafed lupin (*Lupinus angustifolius* L.). Crop and Pasture Science.

[ref-47] Rezania S, Ponraj M, Talaiekhozani A, Mohamad SE, Din MF, Taib SM, Sabbagh F, Sairan FM (2015). Perspectives of phytoremediation using water hyacinth for removal of heavy metals, organic and inorganic pollutants in wastewater. Journal of Environmental Management.

[ref-48] Rojas-Tapias DF, Bonilla R, Dussán J (2014). Effect of Inoculation and Co-inoculation of *Acinetobacter* sp. RG30 and *Pseudomonas putida* GN04 on Growth, Fitness, and Copper Accumulation of Maize (*Zea mays*). Water, Air, and Soil Pollution.

[ref-49] Ryalls JM, Riegler M, Moore BD, Lopaticki G, Johnson SN (2013). Effects of elevated temperature and CO_2_ on above ground–below ground systems: a case study with plants, their mutualistic bacteria and root/shoot herbivores. Frontiers in Plant Science.

[ref-50] Saavedra R, Munoz R, Taboada ME, Vega M, Bolado S (2018). Comparative uptake study of arsenic, boron, copper, manganese and zinc from water by different green microalgae. Bioresource Technology.

[ref-51] Sanchezpardo B, Fernandezpascual M, Zornoza P (2012). Copper microlocalisation, ultrastructural alterations and antioxidant responses in the nodules of white lupin and soybean plants grown under conditions of copper excess. Environmental and Experimental Botany.

[ref-52] Sepulvedacaamano M, Gerding M, Vargas M, Moyaelizondo EA, Oyarzua P, Campos J (2018). Lentil (*Lens culinaris* L.) growth promoting rhizobacteria and their effect on nodulation in coinoculation with rhizobia. Archives of Agronomy and Soil Science.

[ref-53] Seregin IV, Kozhevnikova AD (2006). Physiological role of nickel and its toxic effects on higher plants. Russian Journal of Plant Physiology.

[ref-54] Staudingera C, Mehmeti-Tershania V, Gil-Quintanab E, Gonzalezb EM, Hofhanslc F, Bachmanna G, Wienkoopa S (2016). Evidence for a rhizobia-induced drought stress response strategy in *Medicago truncatula*. Journal of Proteomics.

[ref-55] Tanimoto E (2005). Regulation of root growth by plant hormones-roles for auxin and gibberellin. Critical Reviews in Plant Sciences.

[ref-56] Tashi-Oshnoei F, Harighi B, Abdollahzadeh J (2017). Isolation and identification of endophytic bacteria with plant growth promoting and biocontrol potential from oak trees. Forest Pathology.

[ref-57] Tsang SY, Tam SC, Bremner I, Burkitt MJ (1996). Copper-1, 10-phenanthroline induces internucleosomal DNA fragmentation in HepG2 cells, resulting from direct oxidation by the hydroxyl radical. Biochemical Journal.

[ref-58] Vimal SR, Singh JS, Arora NK, Singh S (2017). Soil-plant-microbe interactions in stressed agriculture management: a review. Pedosphere.

[ref-59] Wani PA, Khan MS, Zaidi A (2008). Effect of metal tolerant plant growth-promoting rhizobium on the performance of pea grown in metal amended soil. Archives of Environmental Contamination and Toxicology.

[ref-60] Yu X, Li Y, Cui Y, Liu R, Chen Q, Gu Y, Zhang X (2017). An indoleacetic acid-producing *Ochrobactrum* sp MGJ11 counteracts cadmium effect on soybean by promoting plant growth. Journal of Applied Microbiology.

[ref-61] Zhang H, Xia Y, Chen C, Zhuang K, Song Y, Shen Z (2016). Analysis of copper-binding proteins in rice radicles exposed to excess copper and hydrogen peroxide stress. Frontiers in Plant Science.

