# Supplementary figures and images for: Promotion of growth and metal accumulation of alfalfa by coinoculation with Sinorhizobium and Agrobacterium under copper and zinc stress
Source: PeerJ. 2019 May 7;7:e6875. doi: 10.7717/peerj.6875 (PMC6510217; doi:10.7717/peerj.6875)

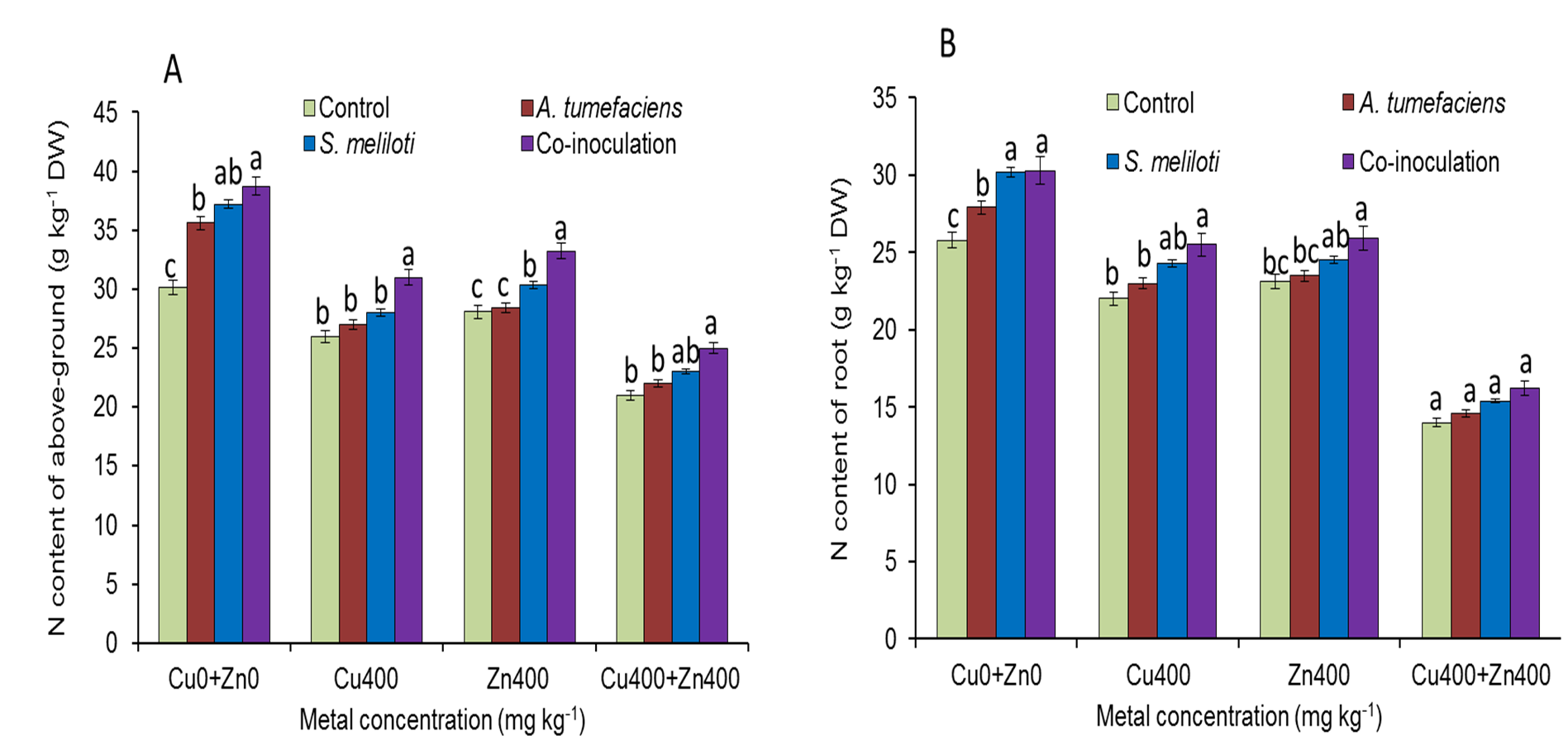

Supplement: Figure S1 — Nitrogen content of the aboveground portion (A) and root (B) of M. lupulina inoculated with S. meliloti or a combination of S. meliloti and A. tumefaciens under moderate (200 mg kg−1) or severe (400 mg kg−1) Cu or Cu (400 mg kg−1) and Zn (400 mg kg−1) double stress conditions. The values indicate the means ± SE of three replicates. Bars carrying different letters denote differences on the basis of a t-test (p < 0.05). [file peerj-07-6875-s001.png]
